# Are Some Countries More Honest than Others? Evidence from a Tax Compliance Experiment in Sweden and Italy

**DOI:** 10.3389/fpsyg.2016.00472

**Published:** 2016-04-07

**Authors:** Giulia Andrighetto, Nan Zhang, Stefania Ottone, Ferruccio Ponzano, John D'Attoma, Sven Steinmo

**Affiliations:** ^1^Robert Schumann Center for Advanced Studies, European University Institute, San Domenico di FiesoleFiesole, Italy; ^2^National Council for Research, Institute of Cognitive Science and TechnologiesRome, Italy; ^3^Department of Social and Political Sciences, European University Institute, San Domenico di FiesoleFiesole, Italy; ^4^Department of Economics, Management and Statistics, University of Milano-BicoccaMilano, Italy; ^5^Department of Law, Politics, Economics and Social Sciences, University of Eastern PiedmontAlessandria, Italy; ^6^Department of Political Science, University of ColoradoBoulder, CO, USA

**Keywords:** tax compliance, ordinary dishonest behavior, fudging, cross-country comparison, social norms

## Abstract

This study examines cultural differences in ordinary dishonesty between Italy and Sweden, two countries with different reputations for trustworthiness and probity. Exploiting a set of cross-cultural tax compliance experiments, we find that the average level of tax evasion (as a measure of ordinary dishonesty) does not differ significantly between Swedes and Italians. However, we also uncover differences in national “styles” of dishonesty. Specifically, while Swedes are more likely to be either completely honest or completely dishonest in their fiscal declarations, Italians are more prone to fudging (i.e., cheating by a small amount). We discuss the implications of these findings for the evolution and enforcement of honesty norms.

## Introduction

Ordinary dishonest behavior rarely attracts much attention. Seemingly innocuous practices such as avoiding VAT, double parking, cheating on an exam, and dodging fares on public transport tend to spread, often even in the wake of high-profile, sensationalized scandals. But while such everyday misdeeds may appear benign, taken together, they can result in vast societal damage (DePaulo et al., 1996; Ariely, 2008; Feldman, 2009; Ayal and Gino, 2011). In this study, we examine cross-national variation in individuals' willingness to engage in ordinary dishonest behavior, as measured by their tendency to underreport income for tax purposes.

The extent to which citizens engage in tax evasion and tax avoidance varies enormously across countries (Schneider and Enste, 2013). This is true even within European nations that share important features such as stable democratic institutions, developed economies, EU membership and broadly similar tax systems. Part of the reason underlying this cross-national variation relates to the efficiency of public institutions. Put simply, countries with efficient institutions (with stringent auditing and financial reporting standards) may be more effective at deterring tax evasion. At the same time, efficient institutions may encourage higher compliance because citizens feel that they are receiving something (i.e., high-quality public services) in return for their money (Levi, 1989; Smith and Stalans, 1991; Smith, 1992; Pommerehne et al., 1994; Edlund, 1999; Frey and Feld, 2002; Frey and Torgler, 2007; Torgler and Schneider, 2007; Cummings et al., 2009; Levi et al., 2009).

However, there is also reason to believe that variation in norms and culture plays an important role in explaining tax evasion. Consider two European countries that arguably lie at opposite ends of the spectrum on tax compliance: Sweden and Italy^1^. Even setting aside differences in the institutional environment, substantial evidence suggests that norms of honesty may differ between these two countries. Specifically, Swedes think that honesty is a typical national trait (Daun, 1989), an assessment shared by other Europeans (Zetterberg, 1995)^2^. By contrast, Italy is ranked very low in terms of honesty amongst European countries, and even Italians themselves consider their compatriots to be less than trustworthy (Mackie, 2001). In fact, the Italian journalist and writer Giuseppe Prezzolini once described Italy as the “country of cunningness” (*paese dei furbi*), where people “worship cunningness so much that they even go so far as to admire those who use it against them” (Prezzolini, 1921).

To what extent can differences in norms and cultures of (dis)honesty explain cross-national variation in fiscal avoidance and evasion? To address this question, we report data from a tax compliance laboratory experiment conducted in Sweden and Italy in 2013/14^3^. Our experimental framework allows us to hold fiscal institutions constant, and thereby isolate the influence of national cultures on individuals' willingness to pay taxes. Given prevailing national stereotypes about norms of dishonesty, we expected that Italians would engage in greater fiscal evasion in the experiment, compared to Swedes.

To preview our basic findings, our experiment reveals, somewhat surprisingly, that *average* levels of tax evasion in Sweden and Italy do not differ significantly. Yet, we uncover country-specific styles of dishonesty. More specifically, we find that Italians engage more frequently in moderately dishonest behavior, or what Ariely (2012) refers to as “fudging.” By contrast, Swedes are more likely to be perfectly honest in their behavior, but among those Swedes who do cheat, they are much more likely to cheat to the maximum extent possible. In the concluding section, we discuss some possible implications of Italians' greater tendency to fudge for the evolution and enforcement of honesty norms, with a particular eye toward explaining Italy's reputation as a “country of cunningness.”

## Related work

Several previous studies have attempted to evaluate cross-national variation in cheating and dishonesty using laboratory experiments. The results have been mixed. On the one hand, a number of studies have found that the propensity to engage in dishonest behavior does not diverge significantly across countries (Gneezy, 2005; Amir et al., 2008; Ariely, 2012; Pascual-Ezama et al., 2015; but see Dieckmann et al., 2015 for contradictory results). On the other hand, when honesty and dishonesty are measured in more real life domains (e.g., tax evasion and bribery scenarios) and framed language is used, systematic and predictable differences are observed across countries (Alm et al., 1995; Torgler, 2004; Bobek et al., 2007; Cummings et al., 2009; Barr and Serra, 2010).

Our study contributes to this literature in two ways. First, we suggest that national or cultural context can influence behavior in the lab under some conditions, but not necessarily others. This is because although honesty norms may differ across societies, normative considerations may have little effect on behavior if not first activated by situational cues in the decision context (Cialdini et al., 1990; Aarts and Dijksterhuis, 2003; Joly et al., 2008). For example, although the general norm in my society may be that “people should not lie,” I could feel perfectly justified in lying to increase my payoffs in a lab experiment, *if* I believe that the operative norm *in that specific context* is to make as much money as possible. Given this, it is unsurprising that experiments using neutral language and context free tasks find little variation in dishonest behavior across countries (since the relevant country-specific norms remain dormant), whereas one finds variation when the specific context is made explicit and the corresponding norms are activated. For this reason, as we describe below, we designed our experiment to explicitly incorporate framed instructions in order to increase the salience of norms against tax evasion^4^.

Secondly, we argue that a consideration of “average” country effects may obscure important variation in patterns of dishonesty. For example, suppose that researchers administer a matrix test to 20 participants, divided evenly between country A and country B. Suppose further that all 10 participants in country A cheat on 50% of the test questions, while in country B, 5 participants are completely honest, while 5 participants are completely dishonest. In this example, “average cheating” is identical across the two countries, but this average also masks important variation in the distribution (i.e., the extent and intensity) of dishonest behavior.

In relation to this last point, several studies have documented heterogeneity in degrees of dishonesty in experimental tasks (Gneezy et al., 2013). More specifically, one general finding emerging from the psychology literature is that, when given opportunities to be dishonest in everyday life, most people are willing to fudge—that is, to cheat “just a little bit” (Mazar et al., 2008; Gino et al., 2009; Ayal and Gino, 2011; Ariely, 2012). The attractiveness of fudging lies in its ability to reduce “ethical dissonance” by allowing people to recast their transgressions in a more benign light, and thereby reconcile dishonesty with the desire to maintain a positive moral self-image (Barkan et al., 2012).

In the context of the foregoing discussion, we are interested in examining how cross-national variation in social norms relating to tax evasion shapes both aggregate tax compliance as well as the tendency to engage in “fiscal fudging.” Accordingly, both of these considerations—norm specificity and average vs. degrees of honesty—inform the design and analysis of the present study.

## Experimental overview

We report results from a tax experiment involving a total of 638 participants in Italy and Sweden (311 in Italy; 327 in Sweden), recruited in five different locations (Rome, Bologna, Milan, Stockholm and Gothenburg)^5^ during the academic year 2013–2014^6^. The basic design of our experiment is similar to that used by Alm (1991), and aims to capture some essential features of the tax system used in many countries: (1) individuals earn real income, (2) they pay taxes on income voluntarily reported, (3) they face some chance that unreported taxes will be detected and penalized, and (4) the total taxes paid are used to provide a public good.

We describe our experimental protocols in detail below, but two features of our methodology are worth highlighting up front. First, our design explicitly provides a “context rich” setting in which tax language is used throughout. This feature is intended to ensure that participants' decisions in the lab reflect their experiences and social norms pertaining to the specific subject under study: taxation (Cummings et al., 2009). By contrast, the standard approach of using neutral language may encourage participants to perceive the decision problem at hand as a risky gamble (i.e., the extra income one earns from unreported taxes weighed against the probability of being caught and fined), as opposed to a tax compliance decision. An additional benefit of framing is that there is no ambiguity for participants about what constitutes honest behavior in the experiment. In other words, unlike in standard public good games in which participants may have different expectations about the appropriate amount of money to contribute, it is clear in the tax frame that the honest behavior is to declare the total amount earned.

Secondly, in our task, participants are not restricted to being either completely honest or completely dishonest, but instead, are allowed to report any amount (from 0 to 100%) of earned income. Thus, our task allows us to test whether Italians and Swedes differ in their tendency to fudge their taxes, an issue that has not been carefully investigated in previous work.

## Experimental protocol

The experiment consisted of four stages, plus a post-experimental survey, and lasted 90 minutes on average. In this article, we report the findings of the first three stages of this experiment^7^. In all, we took great care to ensure that the participant pools were similar in each experimental location^8^, and that the protocol was implemented in exactly the same manner in each country (Appendix Table 1 in the Supplementary Material displays descriptive statistics for each country sample, as well as the degree of similarity between Italian and Swedish participants)^9^.

Each stage began with participants performing a 5 minute clerical task in which they copied random strings of letters and numbers from a sheet of paper onto an electronic form. Participants were paid 10 Experimental Currency Units (ECUs) for each line of text they correctly copied^10^. After the clerical task, participants were shown their earned income and asked to “report your income for tax purposes” under a variety of institutional scenarios (described below). Participants were not informed of how many scenarios would follow or what the specific content of each scenario might be.

In addition, participants were told that they would face a 5% probability of being audited in each scenario; if they underreported their income and were audited, they would pay a fine equal to twice the tax that they had avoided. Importantly, we revealed the results of any audits only at the end of the experiment, to avoid the possibility that being audited in one round would affect behavior in subsequent rounds. Moreover, throughout the experiment, participants had no knowledge of other participants' performance in the typing tasks or their tax reporting decisions. This ensured that individual choices did not reflect reciprocity or conditional cooperation.

In each of the three stages of the experiment, we manipulated fiscal rules relevant to different features of modern taxation systems, in order to elicit behavior under a range of institutional contexts^11^. In stage 1, we altered the amount that participants received in return for the taxes that they collectively paid. In the first scenario (round 1) of stage 1, participants were simply told that the tax rate is 30%. There was no redistribution of tax revenues. In the second scenario (round 2), the tax rate remained 30%, but all tax revenues were placed in a “general fund” which was subsequently divided equally among all participants irrespective of how much each individual paid into the fund. In the third scenario (round 3), we again held the tax rate at 30%, but all tax revenues in the general fund were *doubled* and then redistributed equally to all participants, regardless of how much each participant had individually paid into the fund. In each round (before they were asked to report their incomes), subjects were given multiple specific examples demonstrating the rules in each scenario under a series of hypothetical decisions (see Appendix Supplementary Information 8 in the Supplementary Material); they were also reminded of the 5% probability of being audited, as well as of the fine they would have to pay should the audit detect any under-reporting.

In stage 2, we held redistribution constant and varied the tax rates. In the first scenario of stage 2 (round 4), we asked participants to report their income under a tax rate of 10%. In the second scenario (round 5), the tax rate was increased to 30%, and in the third scenario (round 6), the tax rate was again increased to 50%. In all three rounds of stage 2, we held the audit rate (5%), fines (2x underreported income) and the rules for redistribution (tax revenues doubled and then redistributed) constant.

Finally, in stage 3, we presented scenarios with two different types of progressive taxation schemes. In round 7, the top 10% of income earners (as defined by self reported income) faced a 50% tax rate; participants in the bottom 10% of reported incomes faced a 10% rate; finally, the middle 80% of reported income earners faced a 30% rate. By contrast, in round 8, we introduced a *marginal tax rate system* (similar to the real tax systems operating in Italy and Sweden). In this case, all subjects paid a 10% tax on the first 50 ECUs of reported income, a 30% tax on reported income between 51 and 100 ECUs, and a 50% tax on all reported income above 100 ECUs. In both progressive taxation rounds, all tax revenues were doubled and then redistributed, and we held the audit rate constant at 5%. Once again, subjects were given explicit examples to ensure their understanding of the rules.

## Results

### Average compliance rate

Despite the intrinsic social dilemma structure of the tax scenario that makes evasion the optimal strategy, we find that the level of compliance far exceeded the level predicted by expected utility theory (Allingham and Sandmo, 1972; Yitzhaki, 1974) in both countries and in all rounds. This result is consistent with previous research on tax compliance and public goods (Ledyard, 1995; Bosco and Mittone, 1997; Cummings et al., 2009; Alm, 2012). Pooling both countries, we observe that individuals were mostly honest, reporting on average 64.9% of total income.

**Figure 1:**
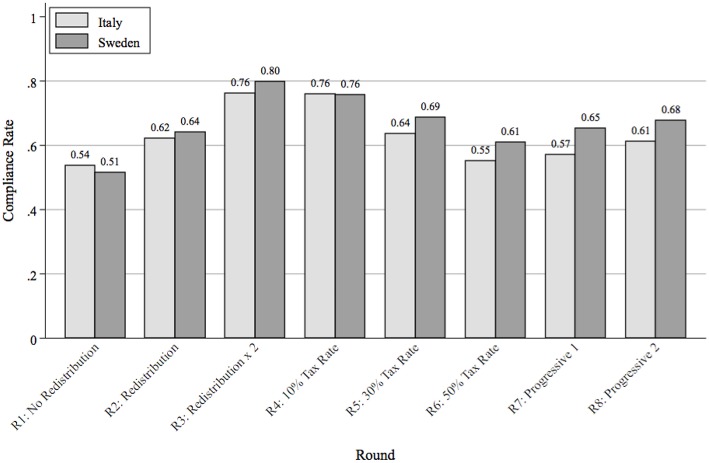
**Average compliance rate divided by round and by country**.

Additionally, we observe that the reporting rate varied according to the specific scenarios presented in each round. Figure 1 shows the average percentage of earned income that was reported in each of the eight rounds, broken down between Swedish and Italian participants. The vertical axis displays the average tax compliance rate, defined as the percentage of total earned income that is truthfully declared in each round. Comparing rounds 1 through 3, we see that compliance responds positively to the efficiency of redistribution: individuals were willing to declare more when they knew that tax revenues produced more public goods. Secondly, individuals responded to higher tax rates by evading their fiscal obligations: compliance falls moving from rounds 4 through 6. These results are in line with previous experimental studies on tax compliance (Alm et al., 1992; Bosco and Mittone, 1997; Torgler, 2002; Blackwell, 2007; Alm, 2012), providing us with some assurance about the validity of our experimental design.

Turning to the cross-country variation in average compliance rates, although we predicted that Swedes would comply more on average than Italians, we do *not* document significant differences across countries. Pooling across all 8 rounds of the experiment, Italians reported 63.1% of their earned income (s.e. = 1.8%), as compared to Swedes who reported 66.6% (s.e. = 1.9%), and the cross-country difference is only 3.5% (*t*-test s.e. = 2.6%, *p* = 0.182). We run several additional tests to assess the robustness of this result. First, we check whether different locations within each country can indeed be pooled to estimate a larger “country” effect. To do so, in Models 1 and 2 of Appendix Table 2 in the Supplementary Material, we estimate individual-level tobit models for the average compliance rate (pooled across all 8 rounds) with site-specific dummy variables, separately for Italy and Sweden^12^. We also cluster standard errors by experimental session. We find no statistically significant within-country variability, suggesting that the results from different locations can indeed be pooled.

Next, we put data from both countries together, and estimate the effect of a dummy variable for Italian participants on the average compliance rate, controlling for a host of individual-level characteristics including gender, age, previous participation in experiments, economics training, earnings in the clerical task, and beliefs about the honesty of other participants. In an alternative specification, we also add fixed effects for the individual treatment round. The inclusion of covariates in Models 3 and 4 of Appendix Table 2 in the Supplementary Material allows us to examine individual-level correlates of tax evasion and dishonesty. We observe that the average compliance rate is lower amongst men, and amongst younger participants (although this latter result is less robust), which is consistent with previous research (Hasseldine, 1999; Lewis et al., 2009; Torgler and Valev, 2010). Risk aversion is also correlated with higher average compliance^13^. In addition, in line with previous work, we find a positive correlation between economics training and lower average compliance (Marwell and Ames, 1981; Carter and Irons, 1991; Cullis et al., 2006; Lewis et al., 2009). Finally, we control for participants' beliefs about the behavior of others in the experiment^14^. Individuals who believed that others reported less also reported less themselves (Fischbacher et al., 2001).

Importantly, the inclusion of these covariates does not change our overall conclusions regarding cross-country differences in the average compliance rate. As shown in Models 3 and 4, the coefficient on the Italy dummy is never statistically significant. These additional results confirm our initial findings reported above: regardless of the controls and model specification we employ, we do not find any significant differences in average compliance rates between the two countries^15^.

### Patterns of compliance and dishonesty

Although an analysis of the *average compliance rate* does not support prevailing national stereotypes that Swedes are more honest than Italians, a closer analysis of the *distribution* of compliance decisions yields some interesting cross-national differences. In particular, a statistic like the average compliance rate does not allow us to distinguish between three different decisions: complete compliance (i.e., the decision to declare 100% of earned income), complete evasion (i.e., the decision to declare 0% of earned income), and partial compliance or “fudging” (i.e., the decision to declare more than 0, but less than the total; see also Mazar et al., 2008 for a similar analysis).

**Figure 2:**
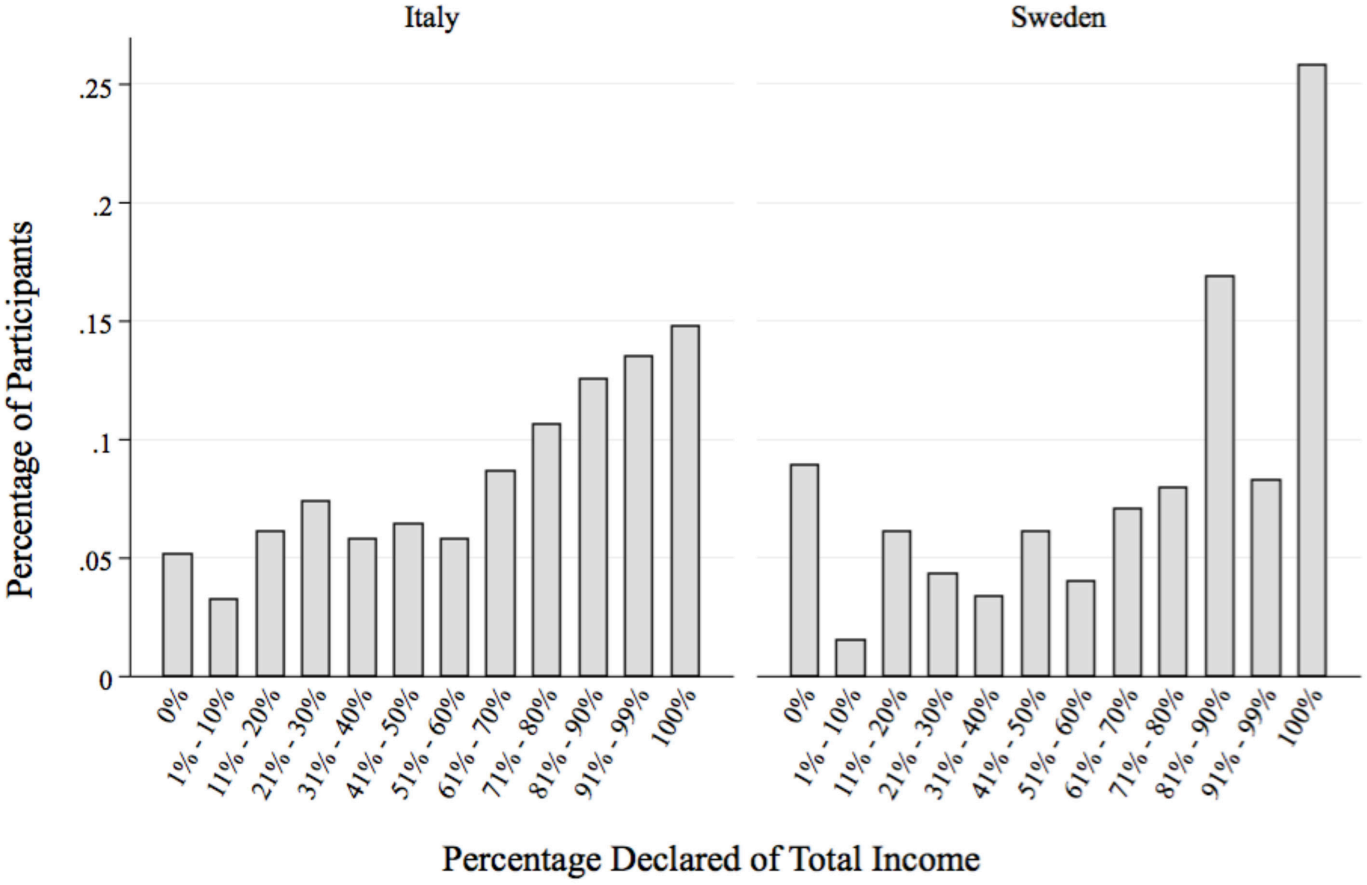
**Distribution of individual compliance rates**.

These distinctions are shown in Figure 2, which displays the distribution of participants' reported incomes (pooled across all 8 rounds). The x-axis breaks down the distribution of reported incomes into the following bins: [0%, (1–10%), (11–20%)…(91–99%), 100%], and the y-axis displays the percentage of participants in each country falling into each bin. We observe that Swedes tended to concentrate in the extreme bins (0% and 100%), while the distribution is more uniform amongst Italians.

To more precisely operationalize these patterns, we define the following three “types” of participants:

- Honest Type: declares 100% of earned income across all 8 rounds.
- Dishonest Type: declares 0% of earned income across all 8 rounds.
- Fudging Type: everyone else.

Next, we compare the distribution of types across Italy and Sweden. We find more Honest Types in Sweden compared to Italy (25.7% in Sweden vs. 14.8% in Italy; Schlag z-test *p* < 0.001), but also more Dishonest Types (8.9% in Sweden vs. 5.1% in Italy; Schlag z-test *p* = 0.066). By contrast, significantly more Italians are classified as Fudging Types (80% in Italy vs. 65% in Sweden; Schlag z-test *p* < 0.001). In other words, Swedish participants displayed more clear-cut behaviors: Swedes cheat less frequently, but when they cheat, they are likely to do so completely. By contrast, Italians cheat more habitually, but the intensity of their cheating is more restrained: they hold back from “cheating all they way.”

Interestingly, we also find that, compared to Dishonest Types, Fudging Types are also more likely to deceive (themselves) about their behavior during the experiment. In particular, in our post-experimental survey, participants were asked to indicate how much of their total earnings they *themselves* reported during the experiment: 18% of Fudging Types indicating that they reported their total income, while no Dishonest Types lied. This last finding nicely fits with evidence from social psychological research showing that individuals choose fudging strategies to maintain a positive moral reputation and self-image (Ayal and Gino, 2011; Ariely, 2012).

To check the robustness of these results, we conduct an additional battery of tests. First, as before, we verify that results from separate locations within countries can indeed be pooled (Models 1 and 2 of Appendix Table 4 in the Supplementary Material)^16^. Next, we estimate probit models of the probability of being a Fudging Type, conditional upon individual-level covariates and round fixed effects (Models 3 and 4 of Appendix Table 4 in the Supplementary Material). In all specifications, Italians were approximately 10% more likely to fudge, compared to Swedes. Here, we also find that individuals who believed that others behaved honestly in the experiment were significantly less likely to fudge^17^^,^^18^.

In summary, although the average *level* of dishonesty does not differ across the two countries, a closer examination of the data reveals a cross-national difference in *patterns* of dishonesty. Simply put: Italians are more prone to “fudging” than Swedes.

## Discussion and concluding remarks

Our results indicate that when Italians and Swedes face a tax compliance scenario consisting of a transparent tax system, efficient redistributive regime, and clear audit rules and penalties, the average level of honesty is relatively high in both countries. This result does not bear out our initial expectations based on national stereotypes, where we predicted a greater level of honesty in Sweden compared to Italy. However, we also identify an interesting cross-country difference that may shed light on our understanding of why these stereotypes emerge. In particular, we find country-specific styles of dishonesty, with Italians engaging more frequently in fudging, while Swedes were more likely to be both perfectly honest and perfectly dishonest. In this concluding section, we offer some conjectures linking this result to the development and perpetuation of national stereotypes about honesty and dishonesty in Sweden and Italy.

In particular, we argue that when ordinary dishonesty takes on the form of fudging, this behavior may be particularly difficult to control and eradicate. Part of the reason stems from the fact that fudging introduces a degree of moral ambiguity in judging the wrongfulness of a particular action. As discussed in Ayal and Gino (2011), when the categorization of a behavior is malleable rather than clear-cut, people are more likely to conceptualize their own actions in acceptable terms. This benevolent interpretation of dishonest behavior helps to reduce any dissonance that may result from the tension between unethical conduct and the desire to maintain a moral self-image (Baumeister, 1998; Schweitzer and Hsee, 2002). Fudging thus provides individuals with greater moral license to indulge in (moderate) wrongdoing.

In addition, in the presence of widespread fudging, it may be difficult for third parties to enforce honesty norms. In particular, when there is uncertainty about what is right or wrong, punishment becomes more risky, since enforcement may generate counter-punishment (also from third-party observers) who do not recognize the legitimacy of the punisher (Herrmann et al., 2008; Strimling and Eriksson, 2014). As such, tolerance for (moderate) wrongdoing rises.

Given the difficulties that fudging poses for both self-regulation and peer-regulation of dishonest behavior, ordinary dishonesty tends to spread. This may explain why Italians have such a widespread reputation for cunningness, as they are observed both to engage in ubiquitous small acts of dishonesty, and to tolerate and even justify dishonesty on the part of others. By contrast, Swedes' relatively clear-cut behaviors may facilitate both self-regulation (as it is more difficult to self-justify gross dishonesty) and social control.

Efforts to raise the moral standard of society in the presence of fudging may thus require actions that (a) increase awareness of the negative effects of apparently benign behaviors, and (b) support norms enforcers who insist on absolute honesty. In future work, we propose to use agent-based modeling and additional experiments to explore the dynamics of fudging, its social effects, and the effectiveness of policy interventions to foster greater public integrity.

## Author contributions

Conceived and designed the experiments: GA, SO, FP, and SS. Performed the experiments: GA, SO, FP, SS, and NZ. Analyzed the data: NZ, SO, and JD. Wrote the paper: GA.

## Funding

The research leading to these results has received funding from the European Research Council under the European Union's Seventh Framework Programme (FP7/2007-2013)/ERC Grant Agreement n. (295675). The funders had no role in study design, data collection and analysis, decision to publish, or preparation of the manuscript.

### Conflict of interest statement

The authors declare that the research was conducted in the absence of any commercial or financial relationships that could be construed as a potential conflict of interest.

## Footnotes

^1^One of the most obvious differences between these two countries is revealed in what is known as the “Tax Gap.” The Tax Gap is a measure of the difference between revenues actually collected and taxes that would have been collected if all taxpayers had honestly reported their incomes. While it is difficult to precisely measure these gaps for obvious reasons, it is widely recognized that the Tax Gap in Sweden is approximately 8–9% of GDP (Slemrod, 2007), whereas in Italy it can reach as high as 25% to 30% (Santoro, 2010).

^2^In a recent YouGov poll, Northern Europeans perceived Sweden as the most honest nation in the EU (YouGov's Eurotrack Series, 2013).

^3^These experiments are part of a larger study on tax compliance behavior in five countries funded by the European Research Council.

^4^However, as noted by an anonymous reviewer, the use of framed instructions could introduce an experimenter demand effect: in particular, participants who wish to “look good” in front of the experimenters may behave more honestly. As we are interested in cross-national differences in behavior, this demand effect would be problematic for our analysis only if it also differs across countries. For example, Italians might care more about “looking good” than Swedes, and thus moderate the amount by which they cheat on their tax declarations in the experiment. However, we do not believe that this possibility poses a serious threat to the validity of our study. In particular, we were careful to ensure from the very beginning that participants had no knowledge that they were taking part in a cross-national comparative study. In other words, there is little reason for Italian (Swedish) participants to feel scrutinized just because they are Italian (Swedish). In addition, we use only native speakers (indeed, in Italy, only native dialect speakers) in each laboratory. This should lessen concerns that one needs to “look good” in front of foreign researchers.

^5^Replicating the experiment in multiple locations within each country provides us with greater confidence that we are not simply picking up “site-specific” effects, but rather cross-country differences in patterns of behavior. We chose these five locations specifically because they were the only active laboratories with suitable characteristics—i.e. with active participant pools drawn from different fields of study — that we could find in Sweden and Italy.

^6^Our experiments have been approved by the IRB Committee at the University of Colorado, Boulder, where the principal investigator holds a professorship. Our project has also been approved by the Ethics Council of the European Research Council, and the European University Institute Ethics committee. Finally, our work has also been authorized by all of the Italian and Swedish laboratories we have used, but we did not undergo a separate university-based IRB review in each case as these were not required by the universities in question. All participants signed a written consent form prior to taking part in the study.

^7^These three stages of the experiment encompass nine rounds of tax reporting (see Appendix Table 7 in the Supplementary Material for a summary). However, we report data from the first 8 rounds only. The 9^th^ round involves donations to a real-world charity, and is not central to our research question. In addition, since the 9^th^ round was the final round (and therefore, did not affect behavior in previous rounds), we have decided to exclude it from the analysis presented in this paper.

^8^Participants were all recruited using ORSEE (Greiner, 2004). In early versions of the experiment, the experimental tasks were programmed and conducted with zTree (Fischbacher, 2007), and the demographic information was collected through Qualtrics. Later in our project, we were able to integrate the experimental and survey portions of the study using our own web-based experimental software. A summary of the reporting rounds and a text version of the instructions (translated into English) are included in Appendix Table 7 in the Supplementary Material and Appendix Supplementary Information 8.

^9^We also had the protocol translated (double-blind) to ensure that the meanings of the words and phrases used were consistent across the countries.

^10^ECUs are converted into real currency at the end of the experiment. One ECU is worth €0.01 in Italy, and 0.60 SEK in Sweden. These exchange rates are chosen based on the average hourly pay rates in each country. The average earnings were 14.09 Euros in Italy and 187.60 SEK in Sweden.

^11^We also considered randomly ordering the scenarios to control for order effects. However, we decided that this option was unnecessary because our central concern is not to evaluate the effects of institutional changes, but rather to examine how people in different countries would respond to the same institutional scenarios.

^12^The number of observations changes once we include demographic covariates in our regression models. This is because in early versions of the experiment, the experimental tasks were implemented in zTree, while the demographic information was collected separately using Qualtrics. This necessitated participants entering their anonymous participant-IDs twice: once into zTree, and once again into Qualtrics. Because some participants accidentally entered different participant-IDs into the two systems, we were unable to match their experimental decisions with their demographic data. This problem was fixed in later versions of the experiment, once we switched to our own web-based experimental software.

^13^We measured risk using a survey item that asks subjects to rank themselves on a 10-point scale, with 1 signifying a person who “normally tries to avoid taking risks” and 10 signifying someone who is “completely willing to take risks.” Answers have been standardized to have mean = 0 and s.d. = 1.

^14^We measured participants' perceptions a survey item which asks subjects whether they thought other participants in the experiment reported (a) their entire earned incomes, (b) less than their entire earned incomes, or (c) much less than their entire earned incomes. In our regressions, we use (b) as our baseline category.

^15^As a further robustness check, we compared country-level differences in average compliance rates separately for each individual round of the experiment. In 6 out of the 8 rounds, we found no statistically significant differences (see Appendix Table 3 in the Supplementary Material).

^16^The percentage of Fudging Types in all Italian locations is higher than in all Swedish locations (83% in Milan, 74% in Bologna and 84% in Rome vs. 67% in Stockholm and 62% in Gothenburg). Running an “empty” random-effects model, we find that the variance within countries is about half the size of the variance across countries.

^17^We also check whether our results are sensitive to the definition of fudging we employ. Specifically, we alternatively redefine Fudging Types as those who reported (a) more than 2/3^rds^ of their income, (b) between 1/3^rd^ and 2/3^rds^ of total income, and (c) less than 1/3^rd^ of total income. Overall, as shown in Appendix Table 5 in the Supplementary Material, we find that regardless of the definition of Fudging Type, Italians were more likely fudge.

^18^We also checked for cross-country differences in the distribution of types separately for each individual round. We find that in all 8 rounds of the experiment, Italians were significantly more likely to fudge than Swedes (See Appendix Table 6 in the Supplementary Material).
