# Supplementary material for: Are Some Countries More Honest than Others? Evidence from a Tax Compliance Experiment in Sweden and Italy
Source: Front Psychol. 2016 Apr 7;7:472. doi: 10.3389/fpsyg.2016.00472 (PMC4823977; doi:10.3389/fpsyg.2016.00472)
Supplement: Supplementary file 1 [file DataSheet1.pdf]

Appendix Table 1: Summary Statistics by Country, and Comparison of Country Means

	Italy			Sweden			Difference in	
	Mean	SD	N	Mean	SD	N	country means	$p < 0.05$?
Percent Male	0.53	0.50	310	0.56	0.50	327	-0.03	No
Age	23.90	3.50	310	27.75	8.45	327	-3.86	Yes
Percent Employed	0.22	0.41	309	0.43	0.50	327	-0.21	Yes
Participated in Experiments Before	0.81	0.39	308	0.73	0.45	327	0.08	Yes
Percent Studying Economics	0.42	0.49	310	0.13	0.34	321	0.29	Yes
Percent Believe: Others Reported…								
Total Income	0.06	0.24	307	0.22	0.41	327	-0.16	Yes
Less than Total Income	0.60	0.49	307	0.66	0.47	327	-0.06	No
Much Less than Total Income	0.34	0.47	307	0.12	0.33	327	0.21	Yes
Willing to Take Risks (std.)	-0.13	1.02	298	0.12	0.96	327	-0.25	Yes
Clerical Task: Rows Copied	34.50	10.74	311	32.79	9.35	327	1.72	Yes

We tested for differences in population means using Schlag's Z-test (for dummy variables) and t-tests (for continuous variables).

Appendix Table 2: Tobit Regression Results: Average Compliance Rate

	Italy	Sweden	Countries Pooled	
	(1)	(2)	(3)	(4)
Italy			0.054	0.176
			(0.030)	(0.104)
Milan	0.040			
	(0.040)			
Rome	0.019			
	(0.044)			
Stockholm		-0.020		
		(0.052)		
Male	-0.198**	-0.272**	-0.235**	-0.626**
	(0.034)	(0.055)	(0.032)	(0.095)
Age (std)	0.057	0.036	0.037	0.131*
	(0.057)	(0.025)	(0.021)	(0.062)
Risk (std)	-0.074**	-0.090**	-0.080**	-0.220**
	(0.028)	(0.032)	(0.019)	(0.048)
Studies Economics	-0.124**	-0.099	-0.112**	-0.319**
	(0.042)	(0.090)	(0.038)	(0.104)
Past Participation	-0.083	-0.098*	-0.091**	-0.239*
	(0.046)	(0.047)	(0.033)	(0.107)
Belief: Total	0.300**	0.220**	0.240**	0.736**
	(0.072)	(0.068)	(0.052)	(0.154)
Belief: Much Less	-0.150**	-0.287**	-0.196**	-0.573**
	(0.045)	(0.073)	(0.040)	(0.113)
Rows Copied	-0.001	-0.003	-0.002	-0.014
	(0.002)	(0.003)	(0.002)	(0.013)
Constant	0.920**	1.057**	0.955**	1.246**
	(0.077)	(0.090)	(0.060)	(0.173)
Round Fixed Effects	-	-	-	Yes
Total Obs.	295	321	616	4925
Left-censored Obs.	15	28	43	1153
Right-censored Obs.	44	84	128	2554
Clusters	Session	Session	Session	Individual
Pseudo r^2	0.250	0.181	0.205	0.104

Clustered standard errors (in parentheses).
** p<0.01, * p<0.05

Appendix Table 3: Tobit Regression Results of Average Compliance, By Round

	R1	R2	R3	R4	R5	R6	R7	R8
Italy	0.318**	0.269*	0.116	0.246	0.112	0.243	0.050	0.078
	(0.121)	(0.132)	(0.143)	(0.185)	(0.115)	(0.139)	(0.111)	(0.083)
Male	-0.843**	-0.766**	-0.468**	-0.542**	-0.589**	-0.655**	-0.606**	-0.492**
	(0.126)	(0.133)	(0.145)	(0.184)	(0.110)	(0.134)	(0.100)	(0.098)
Age (std)	0.188*	0.122	0.079	0.174	0.132	0.189*	0.090	0.083
	(0.075)	(0.075)	(0.093)	(0.109)	(0.070)	(0.086)	(0.081)	(0.052)
Risk (std)	-0.300**	-0.323**	-0.263**	-0.189*	-0.181*	-0.174*	-0.206**	-0.160**
	(0.056)	(0.086)	(0.090)	(0.083)	(0.071)	(0.072)	(0.058)	(0.049)
Studies Economics	-0.142	-0.505**	-0.521**	-0.316	-0.230	-0.443**	-0.286*	-0.233*
	(0.123)	(0.146)	(0.184)	(0.230)	(0.156)	(0.150)	(0.124)	(0.108)
Past Participation	-0.539**	-0.354*	-0.168	0.081	-0.150	-0.331*	-0.186	-0.152
	(0.140)	(0.154)	(0.172)	(0.137)	(0.098)	(0.135)	(0.112)	(0.097)
Belief: Total	0.430*	0.916**	0.948**	0.897**	0.798**	0.944**	0.494*	0.711**
	(0.184)	(0.229)	(0.267)	(0.306)	(0.213)	(0.226)	(0.199)	(0.170)
Belief: Much Less	-0.613**	-0.566**	-0.668**	-0.639**	-0.566**	-0.556**	-0.715**	-0.378**
	(0.123)	(0.180)	(0.205)	(0.238)	(0.134)	(0.185)	(0.135)	(0.094)
Rows Copied	-0.006	0.006	0.036	-0.016	-0.025	-0.001	-0.038**	-0.037**
	(0.013)	(0.017)	(0.021)	(0.028)	(0.019)	(0.021)	(0.012)	(0.012)
Constant	1.440**	1.671**	1.953**	2.241**	1.790**	1.425**	1.933**	1.759**
	(0.192)	(0.231)	(0.264)	(0.423)	(0.291)	(0.316)	(0.198)	(0.190)
Total Obs.	616	616	616	615	615	615	616	616
Left-censored Obs.	217	159	84	96	138	182	155	122
Right-censored Obs.	229	316	427	416	315	278	285	288
Clusters	Session	Session	Session	Session	Session	Session	Session	Session
Pseudo r^2	0.123	0.100	0.0682	0.0479	0.0877	0.0857	0.107	0.109

Clustered standard errors (in parentheses).
** p<0.01, * p<0.05

Appendix Table 4: Probit Results of Fudging

	Italy	Sweden	Countries Pooled	
	(1)	(2)	(3)	(4)
Italy			0.092*	0.084**
			(0.041)	(0.025)
Milan	0.035			
	(0.038)			
Rome	0.060			
	(0.048)			
Stockholm		0.041		
		(0.060)		
Male	0.104*	0.055	0.075*	-0.031
	(0.045)	(0.050)	(0.035)	(0.025)
Age (std)	-0.007	-0.054*	-0.042*	-0.016
	(0.055)	(0.022)	(0.018)	(0.013)
Risk (std)	0.051*	0.023	0.041*	0.018
	(0.025)	(0.021)	(0.018)	(0.012)
Studies Economics	0.105	0.014	0.088	0.038
	(0.059)	(0.079)	(0.047)	(0.027)
Past Participation	0.032	0.051	0.043	-0.067*
	(0.052)	(0.041)	(0.032)	(0.031)
Belief: Total	-0.204	-0.138	-0.137*	-0.117**
	(0.135)	(0.080)	(0.068)	(0.032)
Belief: Much Less	0.021	-0.042	0.014	-0.004
	(0.041)	(0.074)	(0.039)	(0.027)
Rows Copied	0.003*	0.001	0.002	0.000
	(0.001)	(0.003)	(0.002)	(0.004)
Round Fixed Effects	-	-	-	Yes
Obs.	295	321	616	4928
Clusters	Session	Session	Session	Individual
Pseudo r^2	0.109	0.0379	0.0759	0.0464
X^2	55.82	15.82	61.08	133.6

The dependent variable is defined (1 = Fudging Type, 0 = otherwise).
Clustered standard errors (in parentheses).
** p<0.01, * p<0.05

Appendix Table 5: Probit Results of Sensitivity Analysis for Different Definitions of Fudging

	Alternative Definitions of Fudging		
	Report: 1% - 33% (Probit) (1)	Report: 33% - 66% (Probit) (2)	Report: 67% - 99% (Probit) (3)
Italy	0.030*	0.036**	0.014
	(0.012)	(0.013)	(0.014)
Male	0.036**	-0.010	-0.055**
	(0.013)	(0.012)	(0.013)
Age (std)	-0.006	-0.003	-0.007
	(0.006)	(0.007)	(0.008)
Risk (std)	0.008	0.010	-0.001
	(0.007)	(0.006)	(0.006)
Studies Economics	0.006	0.004	0.026
	(0.013)	(0.013)	(0.016)
Past Participation	-0.012	-0.029*	-0.021
	(0.017)	(0.015)	(0.016)
Belief: Total	-0.005	-0.063**	-0.045**
	(0.020)	(0.012)	(0.015)
Belief: Much Less	-0.003	0.007	-0.012
	(0.014)	(0.013)	(0.014)
Rows Copied	-0.004*	0.001	0.003*
	(0.002)	(0.002)	(0.001)
Round Fixed Effects	Yes	Yes	Yes
Obs.	4928	4928	4928
Clusters	Individual	Individual	Individual
Pseudo r^2	0.0338	0.0520	0.0465
X^2	39.44	131.2	72.61

Clustered standard errors (in parentheses).
** p<0.01, * p<0.05

Here, we considered three alternative operationalizations of Fudging Types:

- Low fudging: reporting between 67% - 99% of earned income
- Moderate fudging: reporting between 33% - 66% of earned income
- High fudging: reporting between 1% and 33% of earned income

Appendix Table 6: Complete Compliance, Complete Evasion and Fudging, by Round and by Country

	% Complete Evasion			% Complete Compliance			% Fudging		
	SE	IT	Diff	SE	IT	Diff	SE	IT	Diff
	(1)	(2)	(3)	(4)	(5)	(6)	(7)	(8)	(9)
R1: No Redistribution	0.379	0.318	-0.061	0.404	0.338	-0.066	0.217	0.344	0.127*
			(0.038)			(0.038)			(0.036)
R2: Redistribution	0.266	0.251	-0.015	0.538	0.482	-0.056	0.196	0.267	0.071*
			(0.035)			(0.040)			(0.033)
R3: Redistribution x 2	0.144	0.129	-0.015	0.728	0.653	-0.075*	0.128	0.219	0.090*
			(0.027)			(0.037)			(0.030)
R4: 10% Tax Rate	0.165	0.158	-0.008	0.697	0.640	-0.057	0.138	0.203	0.065*
			(0.029)			(0.037)			(0.030)
R5: 30% Tax Rate	0.226	0.219	-0.008	0.557	0.463	-0.094*	0.217	0.318	0.101*
			(0.033)			(0.040)			(0.035)
R6: 50% Tax Rate	0.303	0.293	-0.010	0.505	0.395	-0.109*	0.193	0.312	0.119*
			(0.036)			(0.039)			(0.034)
R7: Progressive 1	0.245	0.257	-0.013	0.526	0.395	-0.130*	0.229	0.347	0.118*
			(0.034)			(0.039)			(0.036)
R8: Progressive 2	0.205	0.193	-0.012	0.523	0.399	-0.124*	0.272	0.408	0.136*
			(0.032)			(0.039)			(0.037)

We used Schlag's Z-test to test for the difference in country means in terms of the percentage of participants classified as each type. Standard errors displayed (in parentheses).
* indicates whether differences between countries are statistically significant at the 5% level.

Columns (1)-(3) of Table 1 display the proportion of participants who report no income in each round of the experiment. Here, we do not detect any differences between Italians and Swedes in terms of the share of participants who engage in complete evasion. In columns (4)-(6), we show the proportion of participants that report 100% of their earnings for each round. We see that in five out of eight rounds, a significantly higher percentage of Swedes reported their entire income. Finally, the last three columns of Table 1 display the probability of fudging. Here, we uncover the largest cross-national differences in reporting behavior: Italians are more likely to fudge in every single round of the experiment. On average, Italians fudged 2.41 decisions out of 8, compared to Swedes, who

fudged 1.59 decisions (ranksum test p-value< 0.001). We also discover that both Italians and Swedes fudged the most in round 8 (with a progressive flat rate system) where participants have a strong incentive to underreport their earnings to “get under” the 100 ECU threshold.

Appendix Table 7: Summary of Tax Reporting Rounds

Task	Description
Clerical 1	Earn income that is reported in Rounds 1 through 3
Round 1: No Redistribution	Flat tax rate of 30% on all reported income<br><br>Tax revenues are not redistributed
Round 2: Redistribution	Flat tax rate of 30% on all reported income<br><br>Tax revenues are collected into a common fund, which is redistributed on an equal per capita basis to all subjects
Round 3: Redistribution x 2	Flat tax rate of 30% on all reported income<br><br>Tax revenues are collected into a common fund, the amount in the fund is doubled, and then redistributed on an equal per capita basis to all subjects
Clerical 2	Earn income that is reported in Rounds 4 through 6
Round 4: 10% Tax Rate	Flat tax rate of 10% on all reported income<br><br>Tax revenues are collected into a common fund, the amount in the fund is doubled, and then redistributed on an equal per capita basis to all subjects
Round 5: 30% Tax Rate	Flat tax rate of 30% on all reported income<br><br>Tax revenues are collected into a common fund, the amount in the fund is doubled, and then redistributed on an equal per capita basis to all subjects

Round 6: 50% Tax Rate	Flat tax rate of 50% on all reported income<br>Tax revenues are collected into a common fund, the amount in the fund is doubled, and then redistributed on an equal per capita basis to all subjects
Clerical 3	Earn income that is reported in Rounds 7 and 8
Round 7: Progressive 1	Top 10% of earners in Clerical 3 pay 50% tax on reported income<br>Bottom 10% of earners in Clerical 3 pay 10% tax on reported income<br>Everyone else pays 30% tax on reported income
Round 8: Progressive 2	Subjects pay a 10% tax on all reported income under 50 ECU<br>Subjects pay a 30% tax on all reported income between 51 and 100 ECU<br>Subjects pay a 50% tax on all reported income over 100 ECU
Round 9: Charity	Flat tax rate of 30% on all reported income<br>Tax revenues are collected into a common fund, the amount in the fund is doubled, and then proceeds donated to a real-world charity

Appendix Supplementary Information 8: Instructions

WILLING TO PAY?
BASELINE EXPERIMENT INSTRUCTIONS

Below are the instructions given to the subjects both orally and written on their computer screens. The coordinator reads the following aloud as the subjects read along on their computer screens.

- -

SCREEN 1 - ID CODES

Before we begin, please type your experiment ID code into the field on your computer screen. You can find your experiment ID code by opening the envelope we handed to you as you entered the room. If you have any questions about this, please raise your hand now.

Please take a moment to type in your experiment ID code now.

Please press “Continue.”

- -

SCREEN 2 - WELCOME

Welcome. Thank you for agreeing to participate in this experiment.

This experiment will not be particularly difficult or involve trick questions. You will simply need to follow the instructions as they gradually appear on your screen. The answers you will provide will be confidential. Those who process the data from the experiment will not be able to match your name to your choices.

During the experiment, you will be asked to make choices. It is therefore important for the success of the experiment that you do not talk to each other and that you read the instructions very carefully. If you have questions during the experiment, please raise your hand.

At the end of the experiment, you will receive your payment in cash. The actual amount will depend partly on your choices, partly on the choices of the other participants and partly on chance.

Please press “Continue.”

- -

SCREEN 3 – OVERVIEW

Today's experiment is divided into four stages. Every stage is, in all respects, separate from the others. This means that the choices you make in each stage will have no effect on your earnings in the other stages. Your final earnings will be the sum of the earnings obtained in all four stages. At the beginning of each stage, we will read the relevant instructions and give examples to ensure that the experiment is clear.

At the end of the experiment, you will turn in your envelope with your experiment ID number inside. Your earnings will be issued based on this number, not based on your name, so it is important that you retain your ID number. The payments will be issued in cash in the next room, where only one participant at a time may enter. This way none of the other participants will know how much you earned.

Please press "Continue."

- -

SCREEN 4 – THE FIRST THREE STAGES

The first three stages of the experiment are similar to each other in many ways. In each of the first three stages, you will be asked to perform a clerical task for 5 minutes. Based on your performance, you will earn currency units; these will be converted into euros at the end of the experiment. You will participate in several rounds, during which you will be asked to report your income for tax purposes. You are free to report any amount, from 0% to 100% of your earnings. You will only be taxed on the income you report.

There is, however, a 5% probability of being audited. At the end of the experiment, the computer will choose a number between 1 and 100 for each of you in each round. If the result is a number between 1 and 5, you will be audited for that round. If you are audited and you have under-reported your earnings, you will have to pay the tax you should have paid on the non-reported income, plus a fine equal to that non-paid tax. In other words, you will have to pay twice the tax you should have paid on the non-reported income.

Please press "Continue."

- -

SCREEN 5 – CLERICAL TASK

The work that you will complete involves copying data about fictional students from a sheet of paper onto your computer screen, starting from the first line on the list and following the correct order. This piece of paper is in front of you, entitled "Practice Clerical Task." Once you have completed the first line, click the NEXT button. If the computer detects a mistake, you will see an error message, and you will have to correct the mistake before proceeding. If there is no mistake, clicking NEXT will take you to a new screen on which you can start the next line.

For each row copied correctly, you will be paid 10 currency units. Each currency unit in this task is worth 0.01 euros.

We now ask you to try a practice round of this clerical task for 2 minutes. Your performance in this practice round will not count towards your earnings.

Afterward, we will begin the real clerical task, for which you will be paid, and which will last 5 minutes.

Please press "Continue."

. . . .

[verbal instructions only; read between practice and clerical task #1]

Your screen should now show the number of rows you copied correctly. When you have finished viewing your results, please press "Done."

Now that everyone has completed the practice clerical task, we will begin the real clerical task, for which you will earn real currency units that you can later exchange for euros. Please turn to the next page in your packet, entitled "Clerical Task #1." You will start with the first line on this page, which is row number 101.

. . . .

[verbal instructions only; read after clerical task #1]

Your screen should now show the number of rows you copied correctly. When you have finished viewing your results, please press "Done."

- -

SCREEN 6 – TRANSITION SCREEN

Now that everyone has completed the clerical task, you will have to report your income for tax purposes. You will be asked to do this multiple times during the next three rounds.

Please press “Continue.”

- -

SCREEN 7 – INSTRUCTIONS 1.1

In the first round, we ask you to report your income for tax purposes. The tax rate is 30%.

Please remember that you are free to report any amount of your earnings from 0% to 100% for tax purposes. You will only be taxed on the income you report. There is, however, a 5% probability of being audited.

At the end of the experiment, if you are audited and you have under-reported your earnings, you will have to pay the tax you should have paid on the non-reported income, plus a fine equal to that non-paid tax. In other words, you will have to pay twice the tax you should have paid on the non-reported income.

Please press “Continue.”

- -

SCREEN 8 – EXAMPLE 1

Before we begin, let's look at some examples.

Let’s assume that we have three participants: X, Y, and Z. All three participants copy 10 names correctly during the clerical task, and each participant therefore earns 100 currency units.

If Participant X accurately reports his total real earnings of 100 currency units and the tax rate is 30% he will pay 30 in taxes. His final income will therefore be 70 currency units.

If Participant Y reports only 50 currency units, she will pay 15 currency units (since you are only taxed on the income you report). In this case, her final income will be 85 currency units, if she is not audited and fined at the end of the experiment.

If Participant Z reports 0 currency units, he will pay 0 currency units in taxes and his final income will be 100 currency units, unless he is audited and fined at the end of the experiment.

Please press “Continue.”

- -

SCREEN 9 – EXAMPLE 2

What happens if all three participants (X, Y, and Z) are audited and fined at the end of the experiment?

If Participant X is audited, nothing happens: since he reported his total real earnings, he will not incur any penalty, and his final income will be 70 currency units.

If Participant Y is audited, then since she underreported her earnings, she will have to pay twice the tax she did not pay. Since she reported 50 currency units, she owes 15 currency units in taxes. She will therefore have to pay twice the tax she did not pay (30 currency units) and her final income will be 55 (since 85-30=55).

If Participant Z is audited, then since he reported 0 currency units, he owes 30 currency units in taxes. He will therefore have to pay 60 currency units and his final income will be 40 (since 100-60=40).

During the experiment, do not worry about making these calculations. The computer will do them for you.

Please press “Continue.”

- -

SCREEN 10 – REPORTING STAGE

We now ask you to report your income for tax purposes. The tax rate is 30%.

Your earnings from the clerical task are on your screen.

Please report your earnings for tax purposes now.

When you have finished reporting your earnings, please press “Continue.”

- -

SCREEN 11 – INSTRUCTIONS 1.2

The second round is very similar to the first one. As in the first round, we ask you to report your income for tax purposes, at the tax rate of 30%. Again, you are free to report any amount of your earnings (from 0% to 100%) for tax purposes. You will only be taxed on the income you report. There is, however, a 5% probability of being audited. At the end of the experiment, if you are audited and you have under-reported your earnings, you will have to pay the tax you should have paid on the non-reported income, plus a fine equal to that non-paid tax. In other words, you will have to pay twice the tax you should have paid on the non-reported income.
The only difference between the first and the second round is that in the second round, the money that is collected in taxes from all participants will be put into a fund and then redistributed in equal parts among all participants, regardless of how much each participant paid in taxes.

Please press “Continue.”

- -

SCREEN 12 – EXAMPLE

Let's look at an example. Let's assume that 3 people participate in the experiment, and that each one of them earns 100 currency units during the clerical task. One participant reports 100 currency units, one reports 50 currency units, and the other reports 0 currency units. The amount collected in taxes and put into the fund is therefore 45 currency units (since 30+15+0=45). Each participant receives 15 currency units from the fund."

Please press “Continue.”

- -

SCREEN 13 – REPORTING STAGE

We now ask you to report your income for tax purposes. The tax rate is 30%. Please remember that the amount you report in this round is in no way connected to the amount you reported and the tax you paid in the previous round. In other words, your income is exactly the same amount as in the beginning of the first round.

Your earnings from the clerical task are on your screen.

Please report your earnings for tax purposes now.

When you have finished reporting your earnings, please press “Continue.”

- -

SCREEN 14 – INSTRUCTIONS 1.3

The third round is similar to the second one. The only difference is that the amount of money that is collected and put into the fund is now multiplied by 2 before being redistributed in equal parts among all participants. Again, everyone receives an equal amount from the fund, regardless of the amount that each participant paid in taxes.

Please press “Continue.”

- -

SCREEN 15 – EXAMPLE

Let's look at an example.

If the total amount of tax collected is 500 currency units, the amount in the fund will be doubled and the amount redistributed in equal parts among all participants will be 1000 currency units.

Let's look at another example. Let's assume that 20 people participate in the experiment and that each of them pays 100 currency units in taxes. In this case, the amount in the fund will be doubled to 4000 (since 20x100=2000x2=4000) and each participant will receive 200 currency units from it.

Please press “Continue.”

- -

SCREEN 16 – REPORTING STAGE

We now ask you to report your income for tax purposes. The tax rate is 30%. Please remember that the amount you report in this round is in no way connected to the amount you reported and the tax you paid in the previous round. In other words, your income is exactly the same amount as in the beginning of the first round.

Your earnings from the clerical task are on your screen.

Please report your earnings for tax purposes now.

When you have finished reporting your earnings, please press “Continue.”

- -

SCREEN 17 – INSTRUCTIONS 2.0

Now let's begin the second stage of the experiment. The second stage is very much like the first stage. We will again ask you to perform a clerical task for 5 minutes. Based on your performance, you will earn currency units, which will constitute your income in this stage, and which you will be able to turn in for real money at the end of the experiment.

As in the last stage, you will participate in three different rounds, during which we will ask you to report your income for tax purposes. You are free to report any amount (from 0% to 100%) of your earnings. You will only be taxed on the income you report. There is, however, a 5% probability of being audited. At the end of the experiment, if you are audited and it is discovered that you have under-reported your earnings, you will have to pay a penalty equal to twice the tax you should have paid on the income you did not report.

In each round, the money that is collected in taxes from all participants will be put in a fund, multiplied by 2, and redistributed in equal parts among all participants, regardless of how much each participant paid in taxes.

Please press “Continue.”

- -

SCREEN 18 – CLERICAL TASK

We now ask you to perform the clerical task for 5 minutes. Afterwards, you will be told how many currency units you earned. The currency units you earn will constitute your income for the next three rounds.

Please turn to the next page in your packet, entitled “Clerical Task #2.” You will start with the first line on this page, which is row number 201. Once again, we ask you to focus on the clerical task, so that you can earn as much as possible by correctly completing it.

Please press “Continue.”

. . . .

[verbal instructions only; read at the end of the clerical task]

Your screen should now show the number of rows you copied correctly. When you have finished viewing your results, please press “Done.”

- -

SCREEN 19 – TRANSITION SCREEN

Now that everyone has completed the clerical task, we will present you with the different scenarios in which you will have to report your income for tax purposes.

Please press “Continue.”

- -

SCREEN 20 – INSTRUCTIONS 2.1

In the first round, we ask you to report your income for tax purposes. The tax rate is 10%. The money collected in taxes from all participants will be put into a fund, multiplied by 2, and redistributed in equal parts among all participants, regardless of how much each participant paid in taxes

Please remember that you are free to report any amount of your earnings (from 0% to 100%) for tax purposes. You will only be taxed on the income you report. There is, however, a 5% probability of being audited. At the end of the experiment, if you are audited and you have under-reported your earnings, you will have to pay the tax you should have paid on the non-reported income, plus a fine equal to that non-paid tax. In other words, you will have to pay twice the tax you should have paid on the non-reported income.

Please press “Continue.”

- -

SCREEN 21 – REPORTING STAGE

We now ask you to report your income for tax purposes. The tax rate is 10%. Please remember that the amount you report in this round is in no way connected to the amount you reported and the tax you paid in the previous rounds.

Your earnings from the clerical task are on your screen.

Please report your earnings for tax purposes now.

When you have finished reporting your earnings, please press “Continue.”

- -

SCREEN 22 – INSTRUCTIONS 2.2

The second round is similar to the first one. The only difference is that the tax rate is now 30%.

Please press “Continue.”

- -

SCREEN 23 – REPORTING STAGE

We now ask you to report your income for tax purposes. The tax rate is 30%. Please remember that the amount you report in this round is in no way connected to the amount you reported and the tax you paid in the previous rounds. In other words, your income is exactly the same amount as in the beginning of the first round.

Your earnings from the clerical task are on your screen.

Please report your earnings for tax purposes now.

When you have finished reporting your earnings, please press “Continue.”

- -

SCREEN 24 – INSTRUCTIONS 2.3

The third round is very similar to the first and second rounds. The only difference is that the tax rate is now 50%. Please press “Continue.”

- -

SCREEN 25 – REPORTING STAGE

We now ask you to report your income for tax purposes. The tax rate is 50%. Please remember that the amount you report in this round is in no way connected to the

amount you reported and the tax you paid in the previous rounds. In other words, your income is exactly the same amount as in the beginning of the first round.

Your earnings from the clerical task are on your screen.

Please report your earnings for tax purposes now.

When you have finished reporting your earnings, please press “Continue.”

- -

SCREEN 26 – INSTRUCTIONS 3.0

Now let's move on to the third stage of the experiment. The third stage is very similar to the previous ones. We will ask you once again to perform a clerical task for 5 minutes. Based on your performance, you will earn currency units, which will constitute your income in this stage, and which you will be able to turn in for real money at the end of the experiment.

As in the last stage, you will participate in different rounds, during which we will ask you to report your income for tax purposes. You are free to report any amount (from 0% to 100%) of your income. You will only be taxed on the income you report. There is, however, a 5% probability of being audited. At the end of the experiment, if you are audited and you have under-reported your earnings, you will have to pay the tax you should have paid on the non-reported income, plus a fine equal to that non-paid tax. In other words, you will have to pay twice the tax you should have paid on the non-reported income.
Please press “Continue.”

- -

SCREEN 27 – CLERICAL TASK

We now ask you to perform the clerical task for 5 minutes. Afterwards, you will be told how many currency units you earned. The currency units you earned will constitute your earnings in the three rounds of this stage, in which we will present you with different scenarios and ask you to report your income for tax purposes.

Please turn to the next page in your packet, entitled “Clerical Task #3.” You will start with the first line on this page, which is row number 301. Once again, we ask you to focus on the clerical task, so that you can earn as much as possible by correctly completing it.

Please press “Continue.”

. . . .

[verbal instructions only; read at the end of the clerical task]

Your screen should now show the number of rows you copied correctly. When you have finished viewing your results, please press “Done.”

- -

SCREEN 28 – TRANSITION SCREEN

Now that everyone has completed the clerical task, we will present you with the different scenarios in which you will have to report your income for tax purposes.

Please press “Continue.”

- -

SCREEN 29 – INSTRUCTIONS 3.1

In this round, participants will be divided into three groups based on reported income. Those with the highest reported income (the top 10%) will pay a 50% tax rate; those with the lowest reported income (the bottom 10%) will pay a 10% tax rate; and everyone else will pay a 30% tax rate.

In other words, those reporting the highest incomes pay more in taxes, while those reporting the lower incomes pay less. Note, however, that you will not know for sure whether you are a high income, low income, or middle income earner.

As in the previous stage, the money collected in taxes from all participants will be put into a fund, multiplied by 2, and redistributed in equal parts among all participants, regardless of how much each participant paid in taxes.

As in the last stage, you are free to report any amount (from 0% to 100%) of your income for tax purposes. You will only be taxed on the income you report. There is, however, a 5% probability of being audited. At the end of the experiment, if you are audited and you have under-reported your earnings, you will have to pay the tax you should have paid on the non-reported income, plus a fine equal to that non-paid tax. In other words, you will have to pay twice the tax you should have paid on the non-reported income.

Please press “Continue.”

- -

SCREEN 30 – EXAMPLE 1

Let's look at an example. Let's assume that ten subjects participate in the experiment. Of these ten participants, one copies 20 names correctly, one copies 5 names correctly, and the other eight copy 10 names correctly. Every correct name is awarded with 10 currency units. Let's assume, too, that all participants then report their total real earnings for tax purposes.

The participant who copies 20 names correctly has an income equal to 200 currency units and if he reports all 200 currency units pays 50% of it (=100 currency units) in taxes. These 100 currency units are collected in the fund.

The participant who copies 5 names correctly has an income of 50 currency units and if she reports all 50 currency units pays 10% of that income (=5 currency units) in taxes. These 5 currency units are collected into the fund.

Each of the participants who copies 10 names correctly has an income equal to 100 currency units and if he reports all 100 currency units pays 30% of that income (=30 currency units each) in taxes. A total of 240 currency units (8x30) is collected in the fund.

Please press "Continue."

- -

SCREEN 31 – EXAMPLE 2

There are now 345 currency units in the fund (100+240+5=345). The amount collected in the fund is then doubled (345x2=690) and redistributed in equal parts among all participants (690/10=69). Every participant therefore receives 69 currency units from the fund.

In this example, the participant reporting the highest income (200 currency units) pays 100 in taxes, receives 69 from the fund, and therefore has a final income of 169 currency units.

The participant reporting the lowest income (50 currency units) pays 5 in taxes, receives 69 from the fund, and therefore has a final income of 114 currency units.

Each of the other participants (reporting 100 currency units each) pays 30 in taxes, receives 69 from the fund, and therefore has a final income of 139 currency units.

Please press “Continue.”

- -

SCREEN 32 – REPORTING STAGE

We now ask you to report your income for tax purposes. You will pay a tax rate of 50% if your reported income puts you in the top 10% of income earners; a tax rate of 10% if your reported income puts you in the bottom 10% of income earners; and a tax rate of 30% if you are in the middle 80% of reported income earners. Please remember that the amount you report in this round is in no way connected to the amount you reported and the tax you paid in the previous round. In other words, your income is exactly the same amount as in the beginning of the first round of this stage.

Your earnings from the clerical task are on your screen.

Please report your earnings for tax purposes now.

When you have finished reporting your earnings, please press “Continue.”

- -

SCREEN 33 – INSTRUCTIONS 3.2

The second round is very similar to the first one. As in the first round, we ask you to report your income for tax purposes. Again, you are free to report any amount of your earnings (from 0% to 100%) for tax purposes. The probability of being audited at the end of the experiment is, again, 5%. Once again, the money collected in taxes from all participants will be put into a fund, doubled and redistributed in equal parts among all participants, regardless of how much each participant paid in taxes.

The new thing about this round is that you pay 10% for the first 50 currency units of reported income, 30% for any reported income between 51 and 100 currency units, and 50% for any reported income above 100 currency units.

In other words, all participants pay the same tax on the same amount of income, but you pay a different tax rate on different levels of reported income.

Please press “Continue.”

- -

SCREEN 34 – EXAMPLE

Let's look at an example.

Let's assume that Participant X reports 80 currency units for tax purposes. The amount paid in taxes will be equal to 10% on the first 50 currency units, plus 30% on the next 30 currency units, so 14 (=5+9) currency units.

Please press “Continue.”

- -

SCREEN 35 – REPORTING STAGE

We now ask you to report your income for tax purposes. You will pay a tax equal to 10% for the first 50 currency units of reported income, 30% on reported income between 51 and 100 currency units, and 50% on reported income above 100 currency units. Please remember that the amount you report in this round is in no way connected to the amount you reported and the tax you paid in the previous round. In other words, your income is exactly the same amount as in the first round.

Your earnings from the clerical task are on your screen.

Please report your earnings for tax purposes now.

When you have finished reporting your earnings, please press “Continue.”

- -

SCREEN 36 – INSTRUCTIONS 3.3

We now begin the final round. In this round, we ask you to report your income for tax purposes. The tax rate is 30%. As in all the other rounds, you are free to report any amount of your earnings (from 0% to 100%) for tax purposes. You will only be taxed on the income you report. There is, however, a 5% probability of being audited. At the end of the experiment, if you are audited and you have under-reported your earnings, you will have to pay the tax you should have paid on the non-reported income, plus a fine equal to that non-paid tax. In other words, you will have to pay twice the tax you should have paid on the non-reported income.
In this round, however, the money collected in the fund will be multiplied by 2 and the proceeds will be donated to Oxfam [1](note: we really will do this).

[1] UNICEF in Italy

Please press “Continue.”

- -

SCREEN 37 – REPORTING STAGE

We now ask you to report your income for tax purposes. The tax rate is 30%. Please remember that the amount you report in this round is in no way connected to the amount you reported and the tax you paid in the previous rounds. In other words, your income is exactly the same amount as in the beginning of the first round.

Your earnings from the clerical task are on your screen.

Please report your earnings for tax purposes now.

When you have finished reporting your earnings, please press “Continue.”

- -

SCREEN 38 – RESULTS INSTRUCTIONS

The next few screens will reveal your earnings for each stage and each round of the experiment, including whether you were audited and fined for a particular round. You may move through these screens at your own pace; you do not need to wait for permission to move to the next screen.

The final screen will convert your earnings from currency units into euros, so that you can see how much you will be paid for your participation in this experiment.

Please press “Continue” to begin viewing your earnings.

- -

SCREEN 39 – QUESTIONNAIRE INSTRUCTIONS

Finally, we will ask you to complete a short questionnaire, which we will hand out shortly.

Please remain seated and refrain from talking to other participants. Once everyone has completed the questionnaire, we will give you instructions about how to receive your payment.

Appendix Supplementary Information 9: Questionnaire Items

Question	Variable name	Variable type	Answer choices
Age:	Age	integer	- R selects age from dropdown menu
Gender:	Male	integer	Multiple choice radio buttons:<br>0 – Female<br>1 - Male
Are you currently enrolled as a student?	Student	integer	Multiple choice radio buttons:<br>0 – No<br>1 = Yes, undergraduate<br>2 = Yes, masters<br>3 = Yes, PhD or other professional degree
Major field of study<br>(display only if student != 0)	Major	string	- R types the answer
Have you participated in other experiments before?	past_part	integer	Multiple choice radio buttons:<br>0 – No<br>1 = Yes
Do you think most of the participants in the experiment reported their total earnings, less than their total earnings,<br>or much less than their total earnings for tax purposes?	Think_others_report		1=Reported their total earnings<br>2=Reported less than their total earnings<br>3=Reported much less than their total earnings
In the experiment, did you report your total earnings, less than your total earnings, or much less than your total earnings for tax purposes?	Self_report		…
Are you generally a person who is completely willing to take risks, or do you normally try to avoid taking them?<br>Indicate your feelings about this issue	Risk	integer	Row of radio buttons:<br>0 1 2 3 4 5 6 7 8 9 10

below, in which 0 means completely unwilling to take risks, and 10 means completely willing to take risks<br><br>- Completely unwilling (l) vs. Completely willing (r)
